# Bruxism secondary to brain injury treated with Botulinum toxin-A: a case report

**DOI:** 10.1186/1746-160X-2-41

**Published:** 2006-11-23

**Authors:** Mohammed El Maaytah, Waseem Jerjes, Tahwinder Upile, Brian Swinson, Colin Hopper, Peter Ayliffe

**Affiliations:** 1 Oral & Maxillofacial Surgery/Head & Neck Unit, University College London Hospitals, London, UK; 2 Division of Maxillofacial, Diagnostic, Medical and Surgical Sciences, Eastman Dental Institute, 256 Gray's Inn Road, London WC1X 8LD, UK

## Abstract

We report a successful treatment of bruxism in a patient with anoxic brain injury using botulinum toxin-A (BTX-A). On examination the mouth opening was 0 mm, no feeding was possible through the mouth. Botulinum toxin was injected into the masseter and temporalis; great improvement in trismus and bruxism was noted after 3 weeks. One further treatment improved the mouth opening on the following week and the patient was discharged from our care to be reviewed when required.

## Background

The term bruxism is derived from the Greek work "brychein", which means "to grind or gnash the teeth". The reported prevalence is 5 to 96% in adult populations [1-5] and 15% in children [6]. Bruxism is often noted in patients with altered states of consciousness, but its occurrence after brain injury is still unknown. Resolution of bruxism is often associated with improvement in the level of consciousness.

The appearance of bruxism has been closely linked to the return of sleep-wake cycles and improvement of level of consciousness in patients who were initially comatose [7]. To prevent dental wear, mouth guards, spasmolytic medications and relaxation therapy have been used with variable success.

In this report, we describe a successful treatment of bruxism in a patient with anoxic brain injury using botulinum toxin-A (BTX-A).

## Case report

A 26-year-old male suffering global ischemic/hypoxic brain injury after attempted suicide by hanging was admitted to University College London Hospitals (UCLH) intensive care unit for resuscitation following respiratory arrest. The patient remained in coma for 12 days with Glasgow Coma Scale (GCS) of 3–6. Gradual improvement was noticed and the patient started showing signs of alertness and increasing muscle tone of the upper and lower limbs, however they were still in spasm (flexion of upper limbs and extension of lowers). CT scans revealed diffuse low attenuation change in the supratentorial compartment, loss of grey-white matter differentiation and loss of sulcus pattern due to cerebral swelling; a diagnosis of global ischemic/hypoxic brain injury was then reached.

After one month, the patient responsiveness to touch and voice increased, he responded to eye contact and answered questions by yes/no and his GCS reached 15. However, he continued to suffer muscle spasm, have no control over his bladder or bowels and had difficulty in speaking as well as feeding problems; he is currently under multidisciplinary care.

The patient was then referred to the Department of Oral & Maxillofacial Surgery (OMFS), UCLH suffering from trismus and bruxism. Clinical examination revealed a mouth opening of 0 mm; the patient was fed by a Percutaneous Endoscopic Gastrostomy (PEG) tube as oral feeding was impossible. Botulinum toxin was then suggested as a possible treatment for his problem.

**Figure 1:**
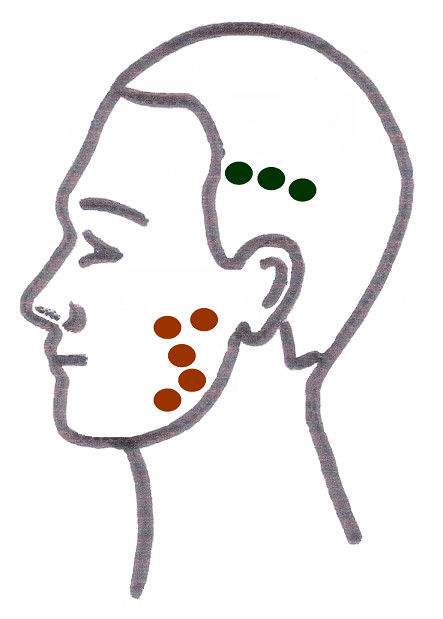
Injection sites in the masseter and temporalis muscles.

The treatment (injections) was carried out in the following visit. Botulinum toxin-A (Botox; Allergan Inc, Irvine, CA) was injected into the right and left masseter and temporalis muscles. One hundred units were reconstituted with 2.5 mL of sterile preservative-free saline and drawn up into an insulin syringe. The skin was cleansed with an alcohol wipe, and the masseter muscle was palpated at its insertion at the angle and body of the mandible. Two injections of 4 Units (2 × 4 U) were given 1 cm superior to the inferior border of the mandible and two other injections of 4 Units (2 × 4 U) were given 1 cm inferior to the inferior border of the zygomatic arch. A fifth injection (1 × 4 U) was given in the centre of the masseter muscle. Three more injections of 4 Units (3 × 4 U) were given 1 cm inferior to the origin of the temporalis muscle. The process was repeated on the contralateral side. (Figure 1)

Three weeks later, the patient was reviewed and had shown a great improvement in his trismus with a mouth opening of 15 mm, with no bruxism reported. There was no erythema, swelling, or any clinical abnormality at the injection sites.

Botox was injected again in the masseter and temporalis muscles 2 weeks later. One week after the second treatment, the patient showed signs of good recovery from trismus and the mouth opening was 20 mm. The patient was discharged from the OMFS care but continued to be under the care of the multidisciplinary team.

## Discussion

Bruxism after brain injury was first described by Pratap-Chand and Gourie-Devi [8]; the group reported that bruxism in comatose patients appeared with the return of sleep-wake cycle; they also suggested that bruxism can occur at varying levels of consciousness and disappear only after a significant improvement in consciousness.

Bruxism has been reportedly caused by, or associated with, numerous conditions such as cranio-cervical dystonia, post-anoxic brain damage, coma, cerebellar damage, Huntington's disease, Rett's syndrome, Whipple's disease, mental retardation and exposure to dopamine receptor-blocking medications as well as selective serotonin re-uptake inhibitors [9]. It has been reported that chewing and teeth grinding can be a useful clinical sign to recognize amphetamine addicts. However, the diagnosis and management of bruxism in addicts have rarely been highlighted [9]. In the head and neck region, successful treatment has been reported for temporomandibular joint (TMJ) disorders, spasmodic torticollis, spastic dysphonia, hemifacial spasm, masseter hypertrophy and "hereditary trembling chin" or Satoyoshi's syndrome [10].

Management options are directed at protecting the teeth by means of readjusting dental occlusion through a variety of intraoral mouth guards, placing stainless steel crowns on teeth or dental extraction [11]. Unfortunately, these interventions are mostly unsuccessful because of the inability of the patients to cooperate with mouth guards or because of the severity of the bruxism.

In 1990, Van Zandijcke and Marchau [12] reported marked reduction in bruxism after injection of botulinum toxin-A into the masseter and temporalis muscles in a patient recovering from a coma.

Muscle weakness (facial weakness) is a known complication for the treatment in this region due to the diffusion of solution across the facial planes [13]; this was not reported by our patient.

Botulinum toxin injection in the masseter muscles is an effective and safe means of intervention in cases of severe post-traumatic bruxism. It may be the only practical intervention available during the period of severe jaw clenching seen after brain injury when the patient is unable to cooperate. Factors that may affect the clinical response include dose, size of the muscles, severity of bruxism and careful identification of the muscles involved.

Trismus after acute cerebrovascular accident (CVA) in adults is a rare complication; however, it was reported by Spillane et al. [14] occurring in an adolescent male; the patient was treated with escalating doses of BTX-A and excellent results were achieved after the third set of injections. The dose used for the third set of injections, given approximately 20 months after CVA, was 300 U total. Recently, Kadyan et al. achieved excellent results using only 10 U of BTX-A in the temporalis muscle [15-17].

We were able to relieve the bruxism and trismus by using 20 U of BTX-A in the masseter muscle per side and 12 U of BTX-A in the temporalis muscle per side; further improvement was noticed after carrying out the treatment one more time.

## References

[B1] Choi YS, Choung PH, Moon HS, Kim SG (2002). Temporomandibular Disorders in 19-Year-Old Korean Men. J Oral Maxillofac Surg.

[B2] Thompson BA, Blount BW, Krumholz TS (1994). Treatment approaches to bruxism. Am Fam Physician.

[B3] Solberg WK, Woo MW, Houston JG (1979). Prevalence of mandibular dysfunction in young adults. J Am Dent Assoc.

[B4] Glaros AG (1981). Incidence of diurnal and nocturnal bruxism. J Prosthet Dent.

[B5] Pavone BW (1985). Bruxism and its effect on the natural teeth. J Prosthet Dent.

[B6] Schneider PE, Peterson J (1982). Oral habits: consideration in management. Pediatr Clin North Am.

[B7] Pratap-Chand R, Gourie-Devi M (1985). Bruxism, its significance in coma. Clin Neurol Neurosurg.

[B8] Pratap-Chand R, Gourie-Devi M (1985). Bruxism: Its significance in coma. Clin Neurol Neurosurg.

[B9] See S-J, Tan E-K (2003). Severe amphetamine-induced bruxism: treatment with botulinum toxin. Acta Neurol Scand.

[B10] Pidcock FS, Wise JM, Christensen JR (2002). Treatment of severe post-traumatic bruxism with botulinum toxin-A: case report. J Oral Maxillofac Surg.

[B11] Attanasio R (1997). An overview of bruxism and its management. Dent Clin North Am.

[B12] Van Zandijcke M, Marchau MM (1990). Treatment of bruxism with botulinum toxin injections. J Neurol Neurosurg Psychiatry.

[B13] Shaari CM (1991). Quantifying the spread of botulinum toxin through muscle fascia. Laryngoscope.

[B14] Spillane KS, Shelton JE, Hasty MF (2003). Stroke-induced trismus in a pediatric patient long-term resolution with botulinum toxin A. Am J Phys Med Rehabil.

[B15] Ivanhoe CB, Lai JM, Francisco GE (1997). Bruxism after brain injury successful treatment with botulinum toxin-A. Arch Phys Med Rehabil.

[B16] Tan EK, Jankovic J (2000). Treating severe bruxism with botulinum toxin. J Am Dent Assoc.

[B17] Winterholler MG, Heckmann JG, Hecht M, Erbguth FJ (2002). Recurrent trismus and stridor in an ALS patient successful treatment with botulinum toxin. Neurology.

